# Brewing By-Products as a Source of Natural Antioxidants for Food Preservation

**DOI:** 10.3390/antiox10101512

**Published:** 2021-09-23

**Authors:** Idoia Codina-Torrella, Lourdes Rodero, María Pilar Almajano

**Affiliations:** 1 Agri-Food Engineering and Biotechnology Department, EEABB, Universitat Politècnica de Catalunya, Esteve Terradas, 8, 08860 Castelldefels, Spain; idoia.codina@upc.edu; 2 Statistics and Operations Research Department, ETSEIB, Universitat Politècnica de Catalunya, Avinguda Diagonal 647, 08028 Barcelona, Spain; lourdes.rodero@upc.edu; 3 Chemical Engineering Department, ETSEIB, Universitat Politècnica de Catalunya, Avinguda Diagonal 647, 08028 Barcelona, Spain

**Keywords:** brewer’s spent grain, spent hop, antioxidant activity, polyphenol, HPLC

## Abstract

Brewer’s spent grain (BSG) and brewer’s spent hops (BSH) are the major solid by-products of the brewing industry. The present work evaluated their potential as an alternative source of natural antioxidants. The efficacy of different solvents (MilliQ water, 0.75% NaOH, 50% MeOH, 50% MeOH + 0.3% HCl and 50% Acetone) for extracting polyphenols of these by-products was firstly evaluated, with NaOH showing the best results. The extraction conditions were optimized using the response surface methodology, and were determined to be 1.45% NaOH and 80 °C. BSG extracts showed the highest total polyphenol content (24.84–38.83 µmol GAE/g), whereas the BSH showed the lowest value (24.84 ± 1.55 µmol GAE/g). In general, BSG extracts presented significantly higher antioxidant capacity (ABTS, ORAC). Ferulic acid was the main polyphenol in all BSG extracts (156.55–290.88 mg/100 g), whereas in BSH, this compound was not detected. The addition of 10% BSG extract in *o*/*w* emulsions (stored 14 days) showed a reduction in the formation of primary oxidation products of 97%. In the emulsions covered with polylactic acid active films (1% BSG), this reduction corresponded to 35%. Hence, this study demonstrates the potential of these by-products as natural antioxidant sources for protecting food systems against oxidation.

## 1. Introduction

Beer is one of the most widely consumed and popular beverages in the world, and is in first place when it comes to the most-consumed alcoholic beverages. The malting and brewing industries are characterized by the generation of large amounts of residues, which account for 85% of their total by-products. In general, these by-products are used as fertilizers, drained into the sewage as waste, or get incinerated [1]. In line with Sustainable Development Goals (SDGs), the food industry is becoming more aware of environmental changes and the negative environmental impacts of their processes and outputs. Sustainable initiatives and enthusiasm for the circular economy have led the brewing industry to reduce its environmental impact through the valorization of its by-products, either by their reduction or their re-incorporation into production processes. These materials are still nutritious, so they can be successfully utilized in various sectors (pharmaceutical, food, biotechnological, etc.).

Brewer’s spent grain (BSG) is the major solid by-product of the brewing industry, and represents around 85% of total residues [2]. This lignocellulosic by-product is obtained after the malt saccharification and lautering processes, and consists of the husks that cover the grains, mixed with other parts of the seed (such as part of the pericarp, coat layers, etc.). BSG is generated at a ratio of ≈20 kg of BSG for every 100 L of produced beer [3]. In general, BSG is commonly considered to be a low-value product, and it is usually mixed with exhausted hops. At present, BSG is widely used in animal feed or as a crop fertilizer, although some studies have also suggested BSG as a potential substrate for biotechnological processes, paper manufacture, or charcoal production [4,5]. The composition of BSG is very heterogeneous, and it is greatly affected by the quality of barley, the presence of other cereals, and the production conditions (such as malting or mashing, among others) [1,6]. Nonetheless, BSG is mainly characterized by its high contents of cellulose (12–25%), hemicellulose (20–25%) and lignin (12–28%), as well as some readily available nutrients such as sugars (~7.5–13.3%), proteins (15.9–35%) and lipids (6.4–13%) [5,7,8,9]. Due to the high moisture content and nutritional composition, BSG has also been demonstrated to be a medium for the cultivation of microorganisms or as a carrier for immobilizing yeast during fermentation processes [1,5]. Recently, different applications in the food industry have also been reported (bakery, confectionary, pasta production, snacks, etc.) [10]. Apart from that, BSG is also a potentially valuable source of low-molecular-weight phenolic compounds [5,7,11], among which polyphenols have attracted a large amount of interest due to their antioxidant properties and their applications in food preservation, and the cosmetic and pharmaceutical sectors [12,13]. However, the effect of BSG supplementation in protecting food against oxidation is still underexploited.

Total phenolic content of BSG is variable (7–10 mg GAE/g d.s.), and comprises mainly bound phenolic compounds [7,8]. Among others, some of the most abundant of these correspond to syringic, ferulic, homovanillic, sinapic and p-cumaric phenolic acids [5,8]. For this reason, the recovery of these compounds is a niche interest in research focused on industrial applications.

Another by-product of the brewing industry is brewer’s spent hops (BSH), which is generated at a ratio of ≈0.2–0.4 kg for every 100 L of produced beer. BSH is often used as a fertilizer or for animal feeding, and its composition is characterized by its contents of lipids (~1%), amino acids, and proteins (~40–52%) [8]. Some authors have reported TPC values in BSH of 10–18 mg GAE/g d.s., and described that their main fraction consists of free polyphenols, such as vanillic, syringic, ferulic and homovanillic acids [8]. The composition of both BSG and BSH is variable, and depends greatly on the raw materials and the brewing processes applied. Different authors have reported the importance of the extraction methods in the optimization of the recovery of these bioactive compounds from BSG and BSH. The most commonly used techniques include conventional solid–liquid extractions (employing water, organic solvents, enzymatic reactions, etc.) [7], in which the structural and compositional features of the matrix and the extraction parameters highly influence the extraction process. Among these methods, extraction with acetone or alkaline hydrolysis has been proved to be the most efficient, and is the most commonly used. However, other emerging extraction techniques have recently been described, such as supercritical extractions and assisted high-pressure, microwave or ultrasound extractions [7,14,15]. Experiments performed with these techniques have also resulted in BSG extracts with high polyphenol content, probably due to the faster heating of the solvent (e.g., in microwave-assisted extractions) or the increase of solvent penetration into the substrate (e.g., in ultrasound-assisted extractions). Nevertheless, in some cases, more research is required to improve and adapt all these extraction mechanisms in order to be able to scale them up.

In line with this, this work aims to investigate the recovery of polyphenols from different artisan and industrial BSG and BSH residues by optimizing the extraction process by using different organic solvents. Response Surface Methodology (RSM) was used to improve the working parameters solvent concentration and extraction temperature. Finally, all extracts were characterized using high-performance liquid chromatography (HPLC) in order to quantify the most abundant phenolic acid. In addition, the feasibility of using BSG extracts as natural antioxidants for food preservation, as well as for application in active packaging, was also investigated.

## 2. Materials and Methods

### 2.1. Raw Material and Chemicals

Five different lyophilized BSG residues (four industrial bagasse (BSG1, BSG2, BSG3 and BSG4), one artisan bagasse (BSG5) and one artisan spent hops (BSH)) were obtained from different local Spanish suppliers.

Reagents were purchased from two different chemical companies. Sigma–Aldrich Ltd. (Gillingham, UK) provided 6-Hydroxy-2,5,7,8-tetramethylchromane-2-carboxylic acid (Trolox), gallic acid, phosphate buffered saline (PBS), 2,2′-azino-bis-(3-ethylbenzothiazoline)-6-sulfonic acid diammonium salt (ABTS), 2,2,-azobis-(2-methylpropionamide) dihydrochloride (AAPH) and fluorescein (C20H10Na2O5), while Panreac (Barcelona, Spain) provided Folin–Ciocalteu reagent, absolute ethanol, aluminum oxide, acetic acid, methanol, ferrous chloride tetrahydrate (FeCl_2_·4H_2_O), ammonium thiocyanate (NH_4_SCN), anhydrous sodium carbonate (Na_2_CO_3_), and Tween 20, all of which were of analytical grade.

### 2.2. Proximate Composition

Protein was determined in BSG extracts (obtained with 1.45% NaOH at 80 °C), with the COBAS analyzer (Roche Diagnostics^®^, Basilea, Switzerland).

For freeze-dried samples, ash content was determined by weight difference before and after incineration at 500 °C for 24 h. Before weighing, samples were placed in a desiccator for 60 min.

All determinations were performed in triplicate.

### 2.3. TPC Extraction and Response Surface Methodology (RSM)

Total phenolic content (TPC) extraction was first optimized by using different solvents (MiliQ water, 0.75% NaOH (*w/v*), 50% MeOH (*v/v*), 50% MeOH + 0.3% HCl (*v/v*) and 50% Acetone (*v/v*)). TPC was determined as described in Section 2.4. For that purpose, industrial BSG3 was used. Preparation of extracts was performed as described by Meneses et al. (2013) [14]. Once the most effective solvent had been selected, the optimal conditions (solvent concentration and temperature) for TPC extraction and radical scavenging activity (ABTS, ORAC) in all BSG samples were obtained by response surface methodology (RSM). Preparation of BSG extracts was performed as described by Meneses et al. (2013) [14]. A two-factor and two-level face-centered central composite design was generated using MINITAB 17 software, with −1, 0, +1 as the coded values of the levels, and considering α = 1. The factors studied corresponded to the temperature and concentration of the solvent, and they were defined according to the following criteria. Conditions were selected considering both, the industrial “real technical possibilities” (corresponding to temperatures ranged between 4 and 80 °C) and solvent concentration values (which were defined according to the previous studies reported in the literature) (Moreira et al., 2012) [16]. With Minitab Software, a 17-run experimental design was obtained, including three replicates of factorial and axial points and 5 replicates of the central point. The experiments were performed in a randomized order to minimize bias effect. Regression coefficients were obtained with a significance level of 0.05. The application of the RSM enables modeling the system to obtain a second-order polynomial model. The generalized second-order polynomial model used in the response surface analysis can be seen in Equation (1), where *Y* corresponds to the response variable, *X_i_* and *X_j_* correspond to independent factors of study, *n* is the number of tested variables, and $\beta_{0},\;\beta_{i},\;\beta_{j}$ and $\beta_{ij}$ represent the coefficients of constant, linear, quadratic and interaction effects, respectively (Equation (1)).

(1) $$Y=\beta_{0}+\overset{j}{\underset{i}{\sum }}\beta_{i}X_{i}+\overset{j}{\underset{i}{\sum }}\beta_{ii}X_{i}^{2}+\overset{j}{\underset{i}{\sum }}\beta_{ij}X_{i}X_{j}$$

The accuracy and reliability of the model were evaluated by analysis of significance (ANOVA), with a 95% of confidence level.

On the basis of the optimized values obtained in the RSM model, TPC and ABTS and ORAC antioxidant activities were then determined (see Section 2.4.). Experimental values were compared with those predicted, validating the model.

### 2.4. Antioxidant Content and Radical Scavenging Activity of Extracts

BSG extracts were analyzed with respect to their radical scavenging activity and antioxidant content. 2,2′-azino-bis-3-ethylbenzo-thiazoline-6-sulphonic acid (ABTS) [17,18] and Oxygen Radical Absorbance Capacity (ORAC) [19] assays were performed. Total phenolic compounds (TPC) [20] and Ferulic acid content [21] were also determined in all samples.

#### 2.4.1. Free Radical Scavenging Activity

The ABTS assay was performed as described by Skowyra et al. (2014) [22]. Absorbance was measured by means of a UV-vis spectrophotometer at 734 nm and the percentage of inhibition was calculated for each sample. The radical scavenging capacity of extracts was quantified as μmol of Trolox equivalent per gram of wet sample (μmol TE/g w.s.).

The ORAC method was adapted from Ou et al. (2001) [23]. The assay was performed with an automated fluorescence microplate reader and 96-well plates, equipped with a temperature-controlled incubation chamber (Fluostar Omega, BMG, Ortenberg, Germany) and incubated at 37 °C. The ORAC value was calculated using a regression equation relating Trolox concentration and the net area under the fluorescence decay curve [23]. Results were expressed as μmol of Trolox equivalents per gram of wet sample (μmol TE/g w.s.).

#### 2.4.2. Total Polyphenol Content

TPC were determined by colorimetric spectrophotometry, according to the Folin–Ciocalteu method and considering the modifications proposed by Segovia et al. (2016) [24]. This assay is based on the ability of a compound to reduce the Folin–Ciocalteu reagent, which resulted in a yellow to green/blue color change. Sample was measured spectrophotometrically at 725 nm, using a UV-vis plate reader spectrophotometer (Fluostar Omega, BMG, Ortenberg, Germany) with gallic acid as standard (10–70 μM). TPC assay is non-specific to phenols and measures any reducing agent. Results are expressed in gallic acid equivalents (GAE) per gram of wet sample (GAE eq/g w.s.).

#### 2.4.3. HPLC-DAD

The content of ferulic acid in the samples was determined following the methodology proposed by Moreira et al. (2012) [16], with some modifications. HPLC system (Waters Alliance 2690, Milford, MA, USA) equipped with a diode array detector (DAD Waters 996) was used. A volume of 20 μL of BSG extract was filtered (0.22 μm) and injected into an analytical C18 column (Synergy Hydro-RP C18, 4 μm, 4.6 × 150 mm, Waters), at 25 °C. The mobile phase was composed of 0.8% acetic acid (*v*/*v*) in water (eluent A) and methanol (eluent B). Elution gradient corresponded to: 90–50% A (minutes 0–25), 50–47% A (minutes 25–75), 47–0% A (minutes 75–95) and 0–90% A (minutes 95–100). Run time was 100 min. and solvent flow rate corresponded to 0.3 mL/min. Detector wavelength was set at 280 nm in order to quantify the ferulic acid, which concentration was calculated using calibration curves. Ferulic acid was used for the external calibration curves. Results are expressed as mg ferulic acid/100 g d.s.

### 2.5. Oxidation Assay: Sample Preparation and Determinations

#### Oil-in-Water Emulsions

Oil-in-water (*o*/*w*) emulsions were prepared according to: 89% MilliQ water, 10% sunflower oil and 1% Tween 20, according to Gallego et al. (2017) [17]. Natural antioxidants of sunflower oil were previously eliminated using activated alumina (Al_2_O_3_) column, as described by Yoshida et al. (1993) [25].

BSG3 extract was prepared under extraction conditions of 1.45% NaOH at 80 °C, as described previously. BSG3 extract was directly mixed in the *o*/*w* emulsions, at a concentration of 10%. These samples were stored in 60 mL amber bottles, in the dark, with constant elliptical movement, and they were allowed to oxidize at 34 ± 1 °C for 14 days. During their storage, the antioxidant capacity of the samples was assessed on the basis of peroxide value determination.

In addition, films with Polylactic acid (PLA films) were also prepared by mixing 3% amorphous PLA (NatureWorks, MW = 75 kDa) with 1% BSG extract, at 40 °C, under constant agitation. Once homogenized, the mixture was decanted and extended onto a glass plate and left to dry for 2 h. Oil-in-water emulsions were also covered with these PLA films, and they were allowed to oxidize under the same conditions as bottled emulsions. PLA films had two sides, which were differentiated in appearance (“smooth” and “rough”), so different emulsions were prepared to test the effect of each side. One sample (bottled or PLA covered) was used in each analysis.

Peroxide value (ferric thiocyanate method) in bottled samples was determined as described by Gallego et al. (2013) [16]. Analyses were performed using a UV-vis spectrophotometer (Fluostar Omega, BMG, Ortenberg, Germany), and the results were expressed as milliequivalents (meq) of hydroperoxide per kg of emulsion.

### 2.6. Statistical Analysis

All tests were performed in triplicate. Results are expressed as means of the triplicates ± standard deviation (SD). Statistical analysis was performed with MINITAB 17 software (Minitab, Inc., State College, PA, USA). Data were analyzed using one-way analysis of variance (ANOVA), and Tukey’s multiple comparison test was used to determine significant differences between samples, with value of *p* < 0.05 being considered significant.

## 3. Results and Discussion

### 3.1. Optimization of Extraction Conditions

#### 3.1.1. Proximate Composition of Extracts

BSG and BSH are two by-products that still contain important amounts of nutritional components (fiber, lipids, protein, minerals, etc.); therefore, many studies have focused on their revalorization in order to integrate them into human food and animal feed [1].

Protein composition of extracts corresponded to BSG1: 20.83 ± 0.75 mg protein/g w.s., BSG2: 24.56 ± 1.13 mg protein/g w.s., BSG3: 22.83 ± 0.28 mg protein/g w.s., BSG4: 21.98 ± 1.00 mg protein/g w.s., BSG5: 8.51 ± 0.11 mg protein/g w.s., BSH: 16.51 ± 0.71 mg protein/g w.s. Proteins in BSG are reported to be around 20% (d.m.), of which hordeins, glutelines, globulins and albumins are one of the most abundant. Approximately 30% of the protein content corresponds to essential amino acids, of which lysine is the most representative [26]. Spent hops content in protein is also high, at ~23% [1].

Ash content in samples was between 1.67 and 3.37% (w.s.). The ash content in the industrial BSG samples was very similar (2.11, 2.94, 2.21, 2.68% w.s. for BSG1, BSG2, BSG3 and BSG4, respectively, whereas the artisan BSG5 exhibited the lowest value (1.67% w.s.). Conversely, BSH presented the highest ash content, corresponding to 3.37% (w.m.), similar to the previous literature [1]. Phosphorous, calcium, magnesium, iron, copper, potassium and manganese are some of the most important minerals of these fractions [1].

#### 3.1.2. Solvent Selection

In the current study, five different solvents were firstly evaluated in order to optimize the extraction of TPC in one of the samples, the BSG3 sample. The most efficient solvent corresponded to 0.75% NaOH, through which 13.24 ± 1.36 µmol GAE/g w.s. was obtained. In line with this, the potential of alkali hydrolysis to favor the release of phenolic acids from rigid lignocellulose structural components has been reported [7]. The TPC of extracts with 50% acetone and 50% MeOH solvents presented the second best results (3.171 ± 0.094 and 1.855 ± 0.134 µmol GAE/g w.s., respectively), with no significant difference (*p* > 0.05) between them. Acetone:water mixtures has also been reported to be one of the most effective solvents for extracting phenolic compounds from different natural sources [14,27], which can probably explain the high efficiency of 50% acetone solvent. Less effective were 50% MeOH + 0.3% HCl and 50% MeOH solvents, neither of which presented differences in TPC recovery (1.45 ± 0.10 and 1.86 ± 0.13 µmol GAE/g w.s., respectively). MilliQ water was the most ineffective solvent for TPC extraction (0.38 ± 0.09 µmol GAE/g w.s.), probably due phenolic compounds often being more soluble in less polar solvents. Therefore, the mixture of water with organic solvents has been widely proposed to facilitate the extraction of these compounds from vegetable sources [28]. Andres et al. (2020) [29], in their study, proposed an efficient extraction methodology for recovering the phenols of BSG by means of using water, but working with longer times (~116–120 min), lower liquid/solid ratios (10–14 mg/mL *v*/*w*) and at lower temperatures (~30 °C). The results obtained in the current study again suggest that the efficiency of phenolic compound extraction is greatly affected by the type of solvent. Therefore, it would be advisable to carry out a cost-effectiveness study, in order to select the most suitable solvents with respect to both aspects: their recovery efficiency and their related production costs.

#### 3.1.3. Optimization Results of Extraction Parameters of Concentration and Temperature through RSM

On the basis of the previous results, sodium hydroxide was selected to proceed with the optimization of TPC extraction though the Response Surface Methodology (RSM). Firstly, the extraction process was optimized to maximize the output variables of TPC, ABTS and ORAC in the BSG3 sample, while varying the input parameters of the solvent concentration (0.05, 0.75, 1.45% (*w/v*)) and the temperature (4, 42, 80 °C) (Table 1). The best TPC was obtained when working with 1.45% NaOH and at 80 °C (30.05 ± 1.31 µmol GAE/g), suggesting that the extraction of TPC was improved when working at higher solvent concentration.

The modification of the polarity of the solvent by increasing the concentration of NaOH changed the solubility of phenolic compounds, consequently improving their recovery (Table 1). This effect was further improved by increasing the extraction temperature (from 4 to 80 °C), which enhanced the solubility and diffusivity of TPC. These two variables showed a significant interaction. Similar results were previously reported by other authors, who extracted TPC of 25.8 ± 0.8 µmol GAE/g when working with 0.75% NaOH at the same temperature [12]. The results observed with ABTS and ORAC assays present a similar trend, in which the best antioxidant activity was obtained working with NaOH at the maximum concentration (1.45%) and at 80 °C. The best antioxidant activity of the BSG3 extract corresponded to 30.05 ± 1.31 µmol GAE/g and 163.73 ± 3.76 µmol TE/g in the ABTS and ORAC tests, respectively.

**Figure 1:**
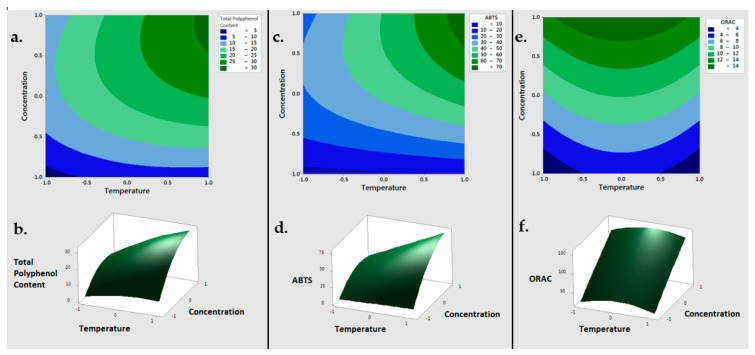
Response Surface Methodology plots in 2D (top) and 3D (bottom) of the Total Polyphenol Content (**a**,**b**), ABTS (**c**,**d**) and ORAC (**e**,**f**) of the BSG sample. ABTS = 2,2′-azino-bis-3-ethylbenzo-thiazoline-6-sulphonic acid, ORAC = 2 oxygen radical absorbance capacity.

Regarding these results, a second-order polynomial model was obtained through the application of the RSM model. Concerning the TPC response, the linear coefficients for NaOH concentration and temperature were positive, whereas their quadratic terms were negative, indicating a positive correlation between TPC and both extraction parameters (solvent concentration and temperature) (Equation (2)). As shown in Figure 1, once the optimal concentration of TPC had been reached, the concentration of the extracted TPC declined slightly.

The maximum value of TPC extraction was not observed in this study, probably because of that point was out of the range proposed for the input variables. The similarity between the average value at the central point (20.989 µmol TE/g w.s.) and the value obtained using the model (21.002 µmol TE/g w.s.) suggest that the model was adequate.

(2) $$\mathrm{TPC}=21.00+6.09T+8.09\left[\mathrm{NaOH}\right]-1.81T^{2}-6.20{\left[\mathrm{NaOH}\right]}^{2}+4.03T\left[\mathrm{NaOH}\right]$$

Analogous results were observed in RSM optimization for ABTS. The antiradical capacity of samples increased when working at higher solvent concentrations and temperatures (Figure 1), reaching an optimal value at 80 °C and 1.45% NaOH (85.49 ± 2.96 µmol TE/g w.s.). As shown for TPC extraction, concentration had a higher impact in ABTS radical scavenging. In this case, the quadratic term of temperature was not significant (*p* > 0.05) (Equation (3)). The similarity between the average value at the central point (43.79 µmol TE/g w.s.) and the value obtained using the model (42.24 µmol TE/g w.s.) suggest that the model possessed good adequacy.

(3) $$\mathrm{ABTS}=42.24+13.19T+21.31\left[\mathrm{NaOH}\right]-12.83{\left[\mathrm{NaOH}\right]}^{2}+11.78T\left[\mathrm{NaOH}\right]$$

Maximum ORAC values (163.72 ± 3.76 µmol TE/g w.s.) were also obtained when working at 80 °C with 1.45% NaOH. In this case, as shown in Equation (4), the concentration of the solvent was the most determining factor, and the temperature appeared to be less relevant. In fact, the second best ORAC value of the analyzed samples (141.98 ± 18.85 µmol TE/g w.s.) was obtained when working with the highest concentration (1.45% NaOH), but at the lowest temperature (4 °C). Furthermore, the antioxidant capacity of the extracts obtained at 42 °C and NaOH concentrations of 0.05 and 0.75% were higher than that observed in their homologs extracted at lower temperatures (Table 1). For NaOH concentration ≤ 1.45%, the maximum ORAC value was obtained when working at 42 °C, but with increasing concentration, the region with the highest antioxidant capacity shifted slightly towards higher temperatures (Figure 1). This corresponds to its appearance as a quadratic term with a negative sign.

(4) $$\mathrm{ORAC}=10.10+5.61\left[\mathrm{NaOH}\right]-2.32T^{2}$$

Discrepancies in the adjustment of the RSM model with respect to the values obtained through the AAPH (ORAC) and ABTS methods could be explained by the differences between the reaction mechanisms. In the ABTS assay, compounds with antiradical capacity neutralize molecules with high steric hindrance, such as the ABTS^•+^ radical [30]. The test sample has to be added after the ABTS^•+^ is generated, and the amount of ABTS^•+^ remaining after reaction with the antioxidants of the sample is measured. Conversely, in the ORAC method, compounds act either directly on peroxyl radicals, or on the radical form of fluorescein. These compounds have a lower steric hindrance, which facilitates the action of antiradical substances. Furthermore, thermal decomposition of antioxidants in ORAC assay due to the effect of temperature (above 42 °C) could also explain these differences [30].

Regarding these results, the RSM model suggested the concentration of sodium hydroxide as the most determining factor for maximize the output variables of TPC, ABTS and ORAC.

### 3.2. Antioxidant Capacity of BSG and BSH Extracts

Once the optimal conditions had been obtained using the RSM model, extractions were performed under the optimized conditions (1.45% NaOH, 80 °C) in all BSG and BSH samples. Table 2 shows the total phenolic content and the radical scavenging activity (TPC, ABTS and ORAC) of these extracts, as well as their ferulic acid content.

Previous studies have described the interesting concentrations of total polyphenols and phenolic acids of both BSG and BSH products [8]. TPC in BSG and BSH extracts ranged from 24.84 to 38.83 µmol GAE/g. Industrial BSG1 extract showed the highest content of TPC (38.83 ± 2.97 µmol GAE/g), although this value was not significantly different (*p* > 0.05) from that obtained in the artisan sample (BSG5) (36.52 ± 0.81 µmol GAE/g). TPC values of other industrial BSG extracts were lower than those observed in BSG1 and BSG6, although these samples did not present differences among them (Table 2). The industrial BSG4 sample (which corresponded to 33.34 ± 1.48 µmol GAE/g) followed the artisan BSG in importance, and showed similar values (*p* > 0.05) for total phenolic compounds to those obtained in the industrial extracts BSG2 and BSG3 (with 30.86 ± 2.46 and 30.15 ± 0.64 µmol GAE/g, respectively). The artisan spent hops extract (BSH) showed the lowest TPC (24.84 ± 1.55 µmol GAE/g) (Table 2). Conversely, Bravi et al. (2021) [8] reported higher total polyphenols content in BSH (~10.90–17.58 mg GAE/g d.m.) than in BSG (~7.30–9.55 mg GAE/g d.m.). These authors also reported that extracted TPC comprised mainly bound phenolic compounds (~70%) in the case of BSG and free phenolic compounds (~90%) in the case of BSH. These results demonstrate again the influence of the origin of these samples on the TPC of these extracts. It is probable that differences in the brewing process, the type of malt, or the hops product used also have an impact on the total phenol content of these extracts. These results suggest BSG to be a sustainable and potential source of natural polyphenols that may be proposed for several applications in food the industry, among others.

Table 2 reports the results obtained for the antioxidant activity of these brewing by-products. In the present study, two different assays were performed (ABTS, ORAC) in order to evaluate the antioxidant capacity of the BSG and BSH extracts. The data showed that the antioxidant activity of the extracts varied according to the sample. The artisan sample (BSG5) exhibited the best antioxidant capacity (90.51 ± 6.41 µmol TE/g and 188.61 ± 11.96 µmol TE/g, for ABTS and ORAC assays), while the artisan spent hops sample (BSH) and the industrial BSG2 sample presented the lowest values (60.03 ± 1.30 µmol TE/g and 152.58 ± 8.24 µmol TE/g and 66.81 ± 3.08 µmol TE/g and 120.18 ± 11.19 µmol TE/g, respectively, for the ABTS and ORAC assays). Industrial BSG1 and BSG4 extracts did not exhibit any differences (*p* > 0.05) in the antioxidant capacity estimated with the ABTS method compared to the artisan extract, although the BSG4 sample showed lower values in the ORAC test. Nevertheless, Bravi et al. (2021) [8] underlined the higher antioxidant power of BSH samples, probably because of these authors worked with extracts with larger amounts total polyphenols. Differences between the antioxidant capacities of the BSG and BSH samples can probably be attributed to their TPC. The best total phenolic compounds contents corresponded to those samples in which the best antioxidant potential was observed.

Birsan et al. (2021) [7] reported ferulic acid as being the most abundant phenolic acid in BSG extracts, representing >50% of the total polyphenol content. Table 2 shows the ferulic acid content of the BSG and BSH extracts, obtained by HPLC-DAD. As shown, this polyphenol was detected in all BSG extracts (156.55–290.89 mg ferulic acid/100 d.s.); however, conversely, this component was not detected in the artisan spent hops sample (BSH) (Table 2). Industrial BSG1 showed the lowest content in that phenolic compound, while industrial BSG3 and BSG2 extracts showed the highest concentration (257.50 ± 17.77 and 290.89 ± 10.15 mg ferulic acid/100 g, respectively) (Table 2). Other authors have also reported salicylic, *p*-coumaric and sinapic acids as other predominant polyphenols in these extracts, followed by homovanillic, syringic, vanillic or caffeic, between others [8,12,31,32]. Conversely, Andres et al. (2020) [29] found that gallic acid accounted for the majority of the TPC extracted of BSG, using water as solvent, whereas sinapic, benzoic and syringic acids were found in lower quantities. These results again suggest that differences observed between samples are in accordance with the characteristics of the raw material, the brewing process, and the extraction method. As shown in Table 3, two strong correlations were observed between the antioxidant activity of samples and their ferulic acid content: TPC and ferulic acid, with a positive correlation (R^2^ = 0.956); and ABTS antiradical capacity and TPC, with a positive correlation (R^2^ = 0.809).

The BSG6 sample was previously eliminated from the correlation analysis, because this component was not detected in that extract. The inverse relationship between ferulic acid and TPC could be attributed to the single phenol group of ferulic acid, which therefore has less relevance than polyphenols in the oxidation–reduction reactions occurring in the Folin–Ciocalteu test. The results showed that the higher the TPC of the samples, the greater the antiradical capacity. Positive correlations between TPC and ABTS have previously been described by other authors (Socaci et al., 2018) [33], who also suggested that the antioxidant capacity of BSG extracts could mainly be attributed to their total polyphenol content.

### 3.3. Food Models: Protection of o/w Emulsions from Oxidation

**Figure 2:**
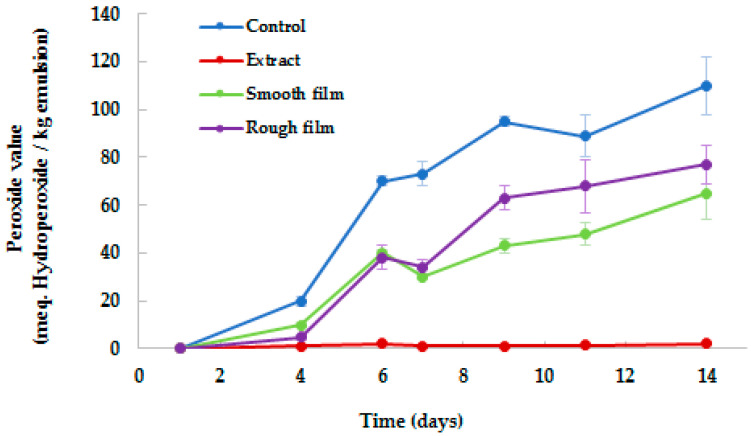
Peroxide value determination of *o*/*w* emulsions protected by BSG. Control = *o*/*w* emulsion; Extract = *o*/*w* emulsion + 10% BSG extract; Smooth film = *o*/*w* emulsion covered with smooth side of PLA film (1% BSG extract), Rough film = *o*/*w* emulsion covered with rough side of PLA film.

Figure 2 shows the evolution in the formation of primary lipid oxidation products, measured as the concentration of hydroperoxides. Free radical-induced oxidation can reduce the nutritional and sensory value and, therefore, the overall quality of fatty-food products.

As observed, BSG extract enhanced the oxidative stability of the *o*/*w* emulsions. If compared to the control (sample without BSG extract), the peroxide values of emulsions with BSG extract remained stable during the 14 days of storage, while in the control, the oxidative deterioration showed a rapid increase. These results indicate that the direct addition of phenolic compounds in *o*/*w* emulsions efficiently protects them (*p* < 0.05) against oxidation, and also supports BSG extracts as a natural source of antioxidants. Simultaneously, the potential of PLA active films for the preservation of *o*/*w* emulsions against the external pro-oxidant agents was also evaluated. After the first 4 days of storage, the control and the emulsions covered with films showed a similar increase in peroxide value, probably due to the low concentration of antioxidants that had been diffused from the film to the emulsions. From that point, the peroxide value in the covered samples differed than that observed in the control, probably due to the gradual release of antioxidants from the film matrix to the emulsions over time, by diffusion, and their activity in trapping the free radicals and chelating metal ions [34]. These results indicate that the storage period has an impact on the antioxidant activity of PLA films. Other authors also observed that the antioxidant activity of films with phenols increased after the films were stored for 6 months, which was also attributed to the polyphenols undergoing polymerization reactions during storage [34]. No differences (*p* > 0.05) were observed between the emulsions covered by smooth and rough sides of PLA film (peroxide value corresponded to 69.26 ± 15.91 μmol TE/g w.s. and 69.26 ± 15.91 μmol TE/g d.w., respectively), demonstrating the antioxidant activity of both sides.

At the end of storage, emulsions with BSG extract showed 97% less oxidation than the control (peroxide value corresponded to 3.35 ± 0.43 μmol TE/g d.w. and 111.37 ± 12.70 μmol TE/g d.w., respectively), while emulsions covered with PLA films exhibited intermediate results. These results again demonstrated the potential of BSG extracts as natural antioxidants, for their direct incorporation in food or for their applications in active packaging. In line with this, in recent years, different studies have promoted industrial and agricultural by-products as potential sources for the extraction of antioxidants. Nowadays, synthetic antioxidants have been used in place of the natural ones, principally due to their higher stability, costs and availability [35]. The interest in these natural components is not only due to their properties and nutritional value, but also due to the economic impact of their revalorization. To date, not many studies have been conducted focusing on biodegradable films with BSG extracts; therefore, this would be also a subject to consider.

## 4. Conclusions

The study carried out shows that BSG and BSH extracts have antioxidant properties. They are residues with a low commercial value, and this article provides evidence that their high percentage of polyphenols allows them to be used as natural antioxidants. Different types of solvent were used to perform the extraction, at different concentrations and temperatures, in order to model the extraction. Solvent concentration was shown to be the most important factor in final performance. The best results were obtained when working with alkaline solvent (NaOH) at a concentration of 1.45% and a temperature of 80 °C.

For this type of extract, the antioxidant capacity is usually proportional to the polyphenol content. This explains why the samples with the highest TPC values were also those with the highest antioxidant activity as determined by the TEAC and ORAC methods with the radicals ABTS and APPH, respectively.

The best results were obtained with an industrial BSG and the artisanal BSG; they showed the highest values of TPC (38.83 ± 2.97 and 36.52 ± 0.81 µmol GAE/g, respectively), while the content in BSH was much lower (24.84 ± 1.55 µmol GAE/g).

Ferulic acid was quantified as one of the main polyphenols in all BSG extracts (156.53–290.89 mg/100 g). It was not detected in BSH, due to the different nature of the compound.

A positive correlation was observed between TPC and radical scavenging activity (ABTS) with ferulic acid content.

In addition, we worked with a model system (oil-in-water emulsion) that made it possible to clearly demonstrate the antioxidant potential for the food industry of BSG extracts and PLA films that incorporate extract and allow controlled release. A total of 10% (*w*/*w*) of the extract was added to the emulsions, and they were stored under forced oxidation conditions for 14 days, allowing a 97% reduction in the formation of primary oxidation products. In the case of emulsions protected with PLA film (which incorporates 1% BSG), the oxidation reduction was 35%, which shows that it is a very promising result that will allow the use of BSA extracts in films active against oxidation. Therefore, this study demonstrates the potential of these by-products as natural sources of antioxidants.

## Figures and Tables

**Table 1:** Results obtained in RSM optimization using industrial BSG 3 sample.

Temperature [°C]	NaOH [%]	TPC [µmol GAE/g w.s.]	ABTS [µmol TE/g w.s.]	ORAC [µmol GAE/g w.s.]
	0.05	3.98 ^e^ ± 0.29	4.87 ^e^ ± 0.11	13.59 ^f^ ± 2.15
4	0.75	11.29 ^d^ ± 0.84	24.91 ^d^ ± 0.53	76.07 ^d^ ± 8.00
	1.45	11.66 ^d^ ± 0.35	31.49 ^d^ ± 2.18	141.98 ^b^ ± 18.85
	0.05	6.32 ^e^ ± 0.46	11.96 ^e^ ± 0.84	47.08 ^e^ ± 1.07
42	0.75	20.99 ^c^ ± 1.31	43.79 ^c^ ± 3.19	103.45 ^c^ ± 7.16
	1.45	23.36 ^b^ ± 1.09	33.83 ^d^ ± 1.35	115.94 ^c^ ± 18.38
	0.05	6.24 ^e^ ± 1.10	7.44 ^e^ ± 0.70	25.16 ^f^ ± 1.08
80	0.75	27.17 ^a^ ± 0.41	51.80 ^b^ ± 3.312	59.21 ^d,e^ ± 0.93
	1.45	30.05 ^a^ ± 1.31	85.49 ^a^ ± 2.96	163.73 ^a^ ± 3.76

*n* = 3. ^a–f^ Values in the same column followed by different superscripts indicate significant differences (*p* < 0.05). TPC = total phenolic content, ABTS = 2,2′-azino-bis-3-ethylbenzo-thiazoline-6-sulphonic acid, ORAC = 2 oxygen radical absorbance capacity, GAE = gallic acid equivalents, TE = trolox equivalents, w.s. = wet sample.

**Table 2:** Characteristics of BSG and BSH extracts.

Sample	TPC [μmol GAE/g w.s.]	ABTS [µmol TE/g w.s.)	ORAC [µmol TE/g w.s.]	Ferulic Acid [mg/100 g d.s.]
Industrial BSG 1	38.83 ^a^ ± 2.97	87.95 ^a,b^ ± 2.12	125.37 ^c,d^ ± 15.76	156.55 ^c^ ± 40.61
Industrial BSG 2	30.86 ^c^ ± 2.46	66.81 ^c,d^ ± 3.08	120.18 ^d^ ± 11.13	257.50 ^a,b^ ± 18.77
Industrial BSG 3	30.15 ^c^ ± 0.64	75.74 ^b,c^ ± 6.28	145.52 ^b,c,d^ ± 1.45	290.89 ^a^ ± 10.15
Industrial BSG 4	33.34 ^b,c^ ± 1.48	87.36 ^a,b^ ± 5.52	165.40 ^a,b^ ± 7.18	217.17 ^b,c^ ± 7.33
Artisan spent hops (BSH)	24.84 ^d^ ± 1.55	60.03 ^d^ ± 1.30	152.58 ^b,c^ ± 8.24	-
Artisan BSG5	36.52 ^a,b^ ± 0.81	90.51 ^a^ ± 6.41	188.61 ^a^ ± 12.00	188.74 ^c^ ± 7.66

*n* = 3. ^a–d^ Values in the same column followed by different superscripts indicate significant differences (*p* < 0.05). TPC = total phenolic content, ABTS = 2,2′-azino-bis-3-ethylbenzo-thiazoline-6-sulphonic acid, ORAC = 2 oxygen radical absorbance capacity, GAE = gallic acid equivalents, TE = trolox equivalents, w.s. = wet sample., d.s. = dry sample.

**Table 3:** Correlations between the TPC, ABTS, ORAC and ferulic acid content.

	TPC ^1^	ABTS ^1^	ORAC ^1^
ABTS	0.81	-	-
ORAC	0.007	0.18	-
Ferulic acid	0.96	0.60	0.03

^1^ TPC = total phenolic content, ABTS = 2,2′-azino-bis-3-ethylbenzo-thiazoline-6-sulphonic acid, ORAC = 2 oxygen radical absorbance capacity.

## Data Availability

The data presented in this study are available in the article.
